# Distribution of XTH, expansin, and secondary-wall-related CesA in floral and fruit abscission zones during fruit development in tomato (*Solanum lycopersicum*)

**DOI:** 10.3389/fpls.2015.00323

**Published:** 2015-05-15

**Authors:** Mutsumi Tsuchiya, Shinobu Satoh, Hiroaki Iwai

**Affiliations:** Faculty of Life and Environmental Sciences, University of Tsukuba, Tsukuba, Japan

**Keywords:** abscission, XTH, expansin, fruit, flower, tomato

## Abstract

After fruit development is triggered by pollination, the abscission zone (AZ) in the fruit pedicel strengthens its adhesion to keep the fruit attached. We previously reported that xyloglucan and arabinan accumulation in the AZ accompanies the shedding of unpollinated flowers. After the fruit has developed and is fully ripened, shedding occurs easily in the AZ due to lignin accumulation. Regulation of cell wall metabolism may play an important role in these processes, but it is not well understood. In the present report, we used immunohistochemistry to visualize changes in the distributions of xyloglucan and arabinan metabolism-related enzymes in the AZs of pollinated and unpollinated flowers, and in ripened fruits. During floral abscission, we observed a gradual increase in polyclonal antibody labeling of expansin in the AZ. The intensities of LM6 and LM15 labeling of arabinan and xyloglucan, respectively, also increased. However, during floral abscission, we observed a large 1 day post anthesis (DPA) peak in the polyclonal antibody labeling of XTH in the AZ, which then decreased. These results suggest that expansin and XTH play important, but different roles in the floral abscission process. During fruit abscission, unlike during floral abscission, no AZ-specific expansin and XTH were observed. Although lignification was seen in the AZ of over-ripe fruit pedicels, secondary cell wall-specific cellulose synthase signals were not observed. This suggests that cellulose metabolism-related enzymes do not play important roles in the AZ prior to fruit abscission.

## Introduction

Abscission is a process by which plants shed unnecessary organs such as leaves, flowers, and fruits to save metabolic energy, to protect themselves from biotic and abiotic stresses by releasing organs attacked by pathogens, and to allow seed dispersal by releasing fruits. Organ abscission occurs at a specialized region in the leaf stem and pedicel, known as the AZ. The AZ consists of several layers of microscopic cells that are distinct from surrounding cells, and which form well before organ separation (Roberts et al., 2000, 2002). Earlier studies have identified several MADS-box genes that are related to the formation of AZs, including *JOINTLESS*, a gene essential for AZ formation in tomato pedicels (Mao et al., 2000). Furthermore, one of the key enzymes in cell wall degradation, polygalacturonase, was first found in the tomato fruit pedicel AZ. Thus, the tomato plant has become an important resource in organ abscission research (Taylor et al., 1991; Kalaitzis et al., 1995, 1997). We previously reported that during floral abscission, a large increase was observed in LM15-labeling of xyloglucan specifically at the AZ in the abscised pedicel. During fruit abscission, unlike in floral abscission, we did not observe any AZ-specific cell wall polysaccharide deposition. However, high autofluorescence was seen in the AZ of overripe fruit pedicels, suggesting secondary cell wall synthesis and lignification of the AZ prior to fruit abscission (Iwai et al., 2013). In the previous report, we did not analyze enzymatic activities of cell wall modeling enzymes, such as xyloglucan endotransglucosylase/hydrolase (XTH), expansin, and certain cell wall synthesis-related enzymes (secondary cell wall enzymes: CesA4, 7, and 8). Biochemical experiments could not be performed due to the limited number of AZ samples, making enzymatic analyses quite difficult. Therefore, in this report, we visualized XTH, expansin, and cellulose synthase using immunohistochemistry.

Xyloglucan is one of the major hemicelluloses of primary cell walls, in dicot plants, and may account for up to 10–20% of cell wall components (Fry, 1989; Hayashi, 1989). They tether cellulose microfibrils by cross-linking them through non-covalent linkages, thus providing strength to the walls during growth. Modification in the length of xyloglucans during cell expansion is primarily mediated by the enzyme XTH through endotransglycosylation, thus enabling the cell wall to expand without weakening (Smith and Fry, 1991; Fry et al., 1992; Nishitani and Tominaga, 1992). XTHs belong to a multigene family (Xu et al., 1996; Campbell and Braam, 1999; Rose et al., 2002; Yokoyama et al., 2004), which plays important roles in several different processes during cell wall modification. These include root hair initiation (Vissenberg et al., 2000, 2001), hypocotyl elongation (Potter and Fry, 1994; Catalá et al., 1997, 2001), hydrolysis of seed storage carbohydrates (de Silva et al., 1993), leaf growth and expansion (Schunmann et al., 1997), aerenchyma formation (Saab and Sachs, 1996), fruit softening (Schroder et al., 1998; Ishimaru and Kobayashi, 2002; Saladié et al., 2006), tension wood formation (Nishikubo et al., 2007, 2011), and petal abscission (Singh et al., 2011). Expansins were the first proteins characterized that directly induce the extension of the plant cell wall (McQueen-Mason et al., 1992), and are believed to be important regulators of wall extension during plant cell growth (reviewed in Lee et al., 2001; Cosgrove et al., 2002; Li et al., 2003). Expansins appear to operate by disrupting hydrogen bonds between cellulose microfibrils and xyloglucans that tether them to one another in plant cell walls (McQueen-Mason and Cosgrove, 1994, 1995; Whitney et al., 2000). In addition to this role during cell growth, expansins play an important role in fruit softening (Rose et al., 1997; Brummell et al., 1999; Anjanasree and Bansal, 2003). Similarly, expansins are expressed at the point of radicle emergence in germinating tomato seeds (Chen and Bradford, 2000) and in the micropylar endosperm of *Datura ferox* seeds in response to red light (Mella et al., 2004), suggesting that they may play a general role in promoting cell wall dissolution. Abscission and fruit softening both involve cell wall breakdown, and many of the same types of enzymes are involved in the two processes (Rose and Bennett, 1999; Rose et al., 2003). Although there is some circumstantial evidence of an association between XTH and expansins and abscission (Cho and Cosgrove, 2000), no reports have been published showing a correlation between the activity of these proteins and organ shedding, especially during floral and fruit abscission.

In the present study, we present the first report that abscission is associated with elevated XTH and expansin, suggesting that these proteins contribute to the process of organ shedding. We also discuss the abscission systems that occur during floral and fruit abscission in tomato plants.

## Materials and Methods

### Plant Material and Growth Conditions

Tomato (*Solanum lycopersicum* cv Micro Tom) plants were grown inside a cultivation chamber (TOMY CL-301) under a 16 h light and 8 h dark regime, at temperatures of 26 and 22°C, respectively, and a light intensity of approximately 100 μmol m^–2^ s^–1^.

### Pollination

Tomato flowers were pollinated by hand. 1 day prior to flowering, the closed buds were opened using a pair of tweezers and the anthers were extracted, leaving only the pistil inside. The opened buds were pollinated the next day by rubbing a dehisced anther onto the stigma. Glassine paper bags were placed over the treated flowers at the time the anthers were extracted to avoid unwanted pollination and to protect against physical stress.

### Technovit Resin Sections

Samples were fixed in 2.5% paraformaldehyde in 0.025 mM phosphate-buffered saline (PBS) and evacuated using a vacuum pump for 12 h. Fixed samples were dehydrated through the following series of EtOH concentrations: 30, 50, 70, 80, and 90% for 20 min each, and then 95 and 100% twice for 30 min. EtOH in dehydrated samples was exchanged for Technovit 7100 resin (Heraeus Kulzer, Wehrheim, Germany) through the following series of Technovit 7100:EtOH: 1:4, 2:3, 3:2, and 4:1 each for 30 min, and then 100% Technovit for 30 min and 12 h. Samples were then solidified in Technovit 7100 resin following the manufacturer’s protocol. Embedded samples were cut into 5 μm sections using a microtome and a glass knife.

### Paraffin Sections

Samples were fixed in 4% paraformaldehyde at 4°C overnight for paraffin embedding. The fixed samples were dehydrated in a graded series of ethanol (70 and 85%) followed by a 1-butanol/ethanol series (80% ethanol/1-butanol 13:7, 90% ethanol/1-butanol 9:11, 100% ethanol/1-butanol 1:3, and 100% 1-butanol). 1-butanol was replaced gradually with paraffin (Paraplast Plus; McCormick Scientific, St. Louis, MO, USA) at 60°C over two nights inside an open jar to evaporate traces of *n*-butanol, and was then embedded in paraffin. Sections 12 μm thick were cut using a rotary microtome (Leica RM2145), and the ribbons were placed and stretched out on albumin-glycerin-treated glass slides with distilled water (DW). The slides were dried at 45°C on a warming plate for 2 days, and stored at room temperature. For use, the slides were deparaffinized in xylene for 10 min (twice), and then hydrated in a graded series of ethanol (100, 90, 80, 70, and 0% in DW).

### Lignin Staining

The sections were soaked in DW prior to staining. Phloroglucinol staining of lignin was performed according to Tadeo and Primo-Millo (1990). A saturated solution of phloroglucinol (Sigma-Aldrich) was prepared in 20% HCl and applied to sections.

### Immunohistochemical Analysis

Monoclonal rat IgG antibodies of LM6 and LM15 were purchased from PlantProbes (Leeds, UK^1^). Anti XTH, CesA4, CesA7, and CesA8 rabbit antibodies were obtained from Agrisera (Vännäs, Sweden ^2^^3^^4^^5^). Anti expansin antibody was obtained from Biocompare (South San Francisco, San Mateo, CA, USA^6^). A TSA kit with HRP-conjugated secondary antibody and Alexa Fluor 488 tyramide were purchased from Invitrogen (Carlsbad, CA, USA; cat. #T20912, #20922, respectively). We used the set of polyclonal antibodies to perform immunohistochemistry according to the manufacturer’s instructions. Briefly, the sections were incubated in PBS prior to labeling, and 100 μL of the following reagents were added to the sections in consecutive order: quenching buffer (to quench endogenous peroxidase activity), 1% blocking reagent, and primary antibody diluted in 1% blocking reagent (1:30). After each addition, the sections were incubated at room temperature for 1 h. The sections were then washed three times with PBS, incubated in 100 μL horseradish peroxidase (HRP)-conjugated second antibody diluted in 1% blocking reagent (1:100) for 1 h, again washed three times with PBS, incubated in 100 μL Alexa Fluor 488 tyramide (excitation 495 nm, emission 519 nm) working solution (tyramide stock solution diluted in amplification buffer/0.0015% H_2_O_2_; 1:100) for 10 min at room temperature, and finally washed three times with PBS and twice with DW. Immunofluorescence was visualized using a Leica DMRB fluorescence microscope, and all of the micrographs were captured with a DFC500 Leica digital camera using the IM50 Leica software (exposure time, 1.0 s).

## Results

### Immunolocalization of Xyloglucan and Arabinan Epitopes During –1 DPA Flower Pedicels to Yellowing Pedicels

**FIGURE 1:**
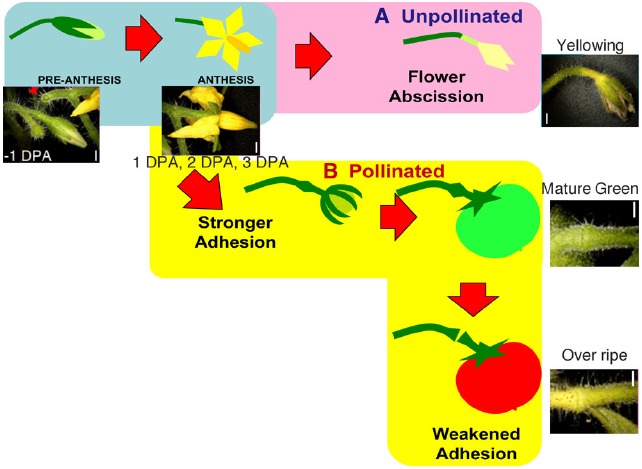
**Schematic diagram of the regulation of flower and fruit adhesion in tomato AZs.** Once the flowers are successfully pollinated and fruits start to develop, the AZs enlarge and adhesion becomes stronger until the fruits fully ripen **(B)**. Unsuccessful pollination leads to flower detachment from the AZ **(A)**. Red arrowheads indicate pedicels separated at AZ. Bars = 1 mm. Adapted from Iwai et al. (2013).

To investigate the distribution of cell-wall pectic arabinan and xyloglucan, we used their specific monoclonal antibodies to stain –1 DPA, 1 DPA, 2 DPA, 3 DPA pedicels and yellowing pedicels (Figure 1). Pedicels yellowed just before abscission. Immunodot assays and competitive inhibition ELISA performed by Willats et al. (1998) and Orfila and Knox (2000) indicated that the LM6 antibody is specific to (1 → 5)-α-L-linked arabinosyl residues with extended polymer, and reacts with rhamnogalacturonan (RG)-I but not RG-II. ELISA and glycan microarray performed by Marcus et al. (2008) indicated that LM15 binds specifically to xyloglucan, and competitive inhibition ELISAs determined that the structural feature for LM15 binding is the XXXG structural motif. Therefore, we used these probes to explore the occurrence of specific cell wall polysaccharides and differences in their distribution during early fruit development.

**FIGURE 2:**
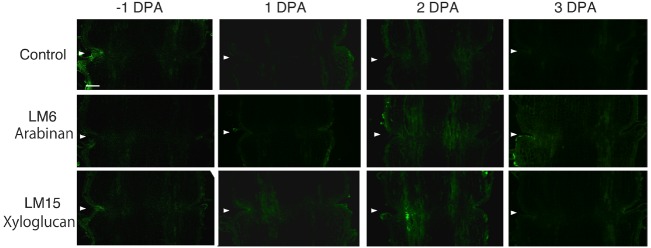
**Immunolocalization of arabinan and xyloglucan epitopes in the AZs of –1 DPA to 3 DPA pollinated flower pedicels.** Pollination allowed the pedicel and AZ to enlarge and strengthen in adhesion. Longitudinal sections of –1 DPA flower pedicels, including the AZ, were stained with the antibodies LM6 (anti-arabinan) and LM15 (anti-xyloglucan). Micrographs show the negative control without the first antibody step. Arrowheads indicate the AZs. Bar = 0.2 mm.

In the pollinated flower pedicel, the intensity of LM15 xyloglucan epitopes was low in –1 DPA to 3 DPA. The intensity of LM6 arabinan and LM15 xyloglucan epitopes was low throughout the tissue (Figure 2). Some weak signals were detected in vascular bundles by LM15; however, the labeling was not specific to the AZ.

**FIGURE 3:**
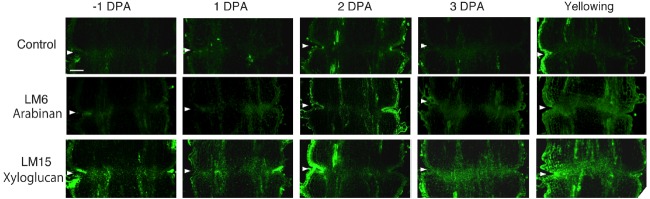
**Immunolocalization of arabinan and xyloglucan epitopes in the AZs of –1 DPA to yellowing unpollinated flower pedicels.** Unsuccessful pollination led to flower abscission. Longitudinal sections of –1 DPA flower pedicels, including the AZ, were stained with the antibodies LM6 (anti-arabinan) and LM15 (anti-xyloglucan). Micrographs show the negative control without the first antibody step. Arrowheads indicate the AZs. Bar = 0.2 mm.

In unpollinated flower pedicels, relative to –1 DPA pedicels, the intensity of LM6 pectic arabinan labeling increased in 3 DPA, and the highest signal was detected in the yellowing stage (Figure 3). The LM6 labeling was most intense at the AZ (Figure 3). Remarkably, LM15 labeling for xyloglucan epitopes increased dramatically, preferentially at the AZ (Figure 3). These results indicate that pectic arabinan and xyloglucan increase prior to abscission. As the time of abscission approached, accumulation of xyloglucan and arabinan increased (Figure 3).

### Immunolocalization of Expansin and XTH Epitopes During –1 DPA Flower Pedicels to Yellowing Pedicels

**FIGURE 4:**
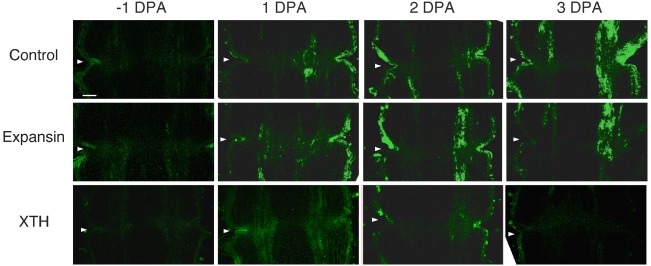
**Immunolocalization of expansin and XTH epitopes in the AZs of –1 DPA to 3 DPA pollinated flower pedicels.** Pollination allowed the pedicel and AZ to enlarge and strengthen in adhesion. Longitudinal sections of –1 DPA flower pedicels, including the AZ, were stained with the anti-expansin and anti-xyloglucan antibodies. Micrographs show the negative control without the first antibody step. Arrowheads indicate the AZs. Bar = 0.2 mm.

**FIGURE 5:**
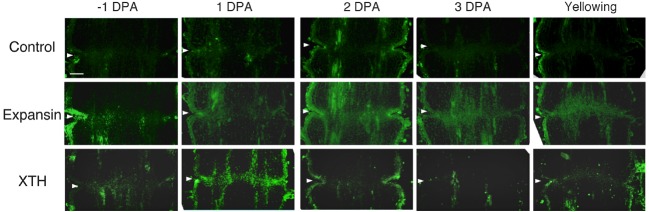
**Immunolocalization of expansin and XTH epitopes in the AZs of –1 DPA to yellowing unpollinated flower pedicels.** Unsuccessful pollination led to flower abscission. Longitudinal sections of –1 DPA flower pedicels, including the AZ, were stained with the anti-expansin and anti-xyloglucan antibodies. Micrographs show the negative control without the first antibody step. Arrowheads indicate the AZs. Bar = 0.2 mm.

To investigate the potential role of XTH and expansin during the time course of floral abscission, we visualized changes in the distributions of XTH and expansin epitopes in –1 DPA, 1 DPA, 2 DPA, 3 DPA pedicels and yellowing pedicels. XTH was determined by immunolocalization using polyclonal antibodies (Liu et al., 2013). In tomatoes, *SlXTH1–25* was reported as the gene that encodes XTH (Muñoz-Bertomeu et al., 2013) and the antibody detected epitopes in all the XTH proteins examined (Takizawa et al., 2014). Expansin is a peripheral membrane protein that may cause loosening and extension of plant cell walls by disrupting non-covalent bonding between cellulose microfibrils and matrix glucans. Tomato expansins have also been reported (Rose et al., 2000), and we used commercial polyclonal antibodies that are reactive to β-expansin as shown in web page of antibody-online.com^7^. The epitope(s) recognized by the anti-β-expansin is thought to occur in all tomato expansins. In the pollinated flower pedicel, the intensities of XTH and expansin epitopes were low in –1 DPA to 3 DPA (Figure 4). In the unpollinated flower pedicel, LM6 and LM15 labeling of arabinan and xyloglucan increased (Figure 3). However, during floral abscission, we observed a large 1 DPA peak in the polyclonal antibody labeling of XTH in the AZ, which then decreased (Figure 5).

Remarkably, expansin epitopes increased gradually but dramatically, specifically at the AZ, during floral abscission, whereas LM6 and LM15 labeling of arabinan and xyloglucan, respectively, also increased (Figure 3). The highest signals of expansin were detected during the yellowing stage just prior to abscission (Figure 5)

### Immunolocalization of XTH and Expansin Epitopes in MG Fruit Pedicels and OR Fruit Pedicels

**FIGURE 6:**
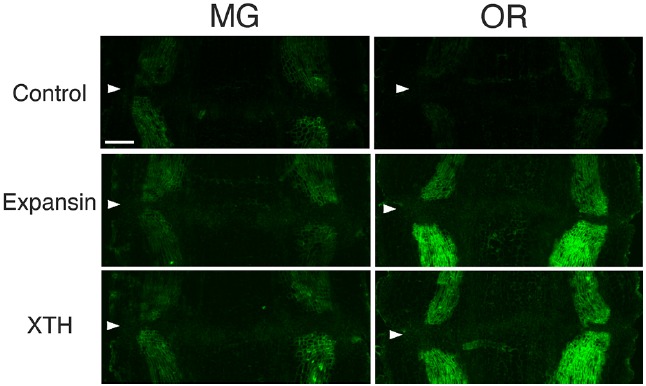
**Immunolocalization of expansin and XTH epitopes in the AZs of mature green (MG) and over-ripe (OR) fruit pedicels.** Longitudinal sections of MG fruit pedicels, including the AZ, were stained with the anti-expansin and anti-xyloglucan antibodies. Micrographs show the negative control without the first antibody step. Arrowheads indicate the AZs. Bar = 0.5 mm.

To determine whether flower and fruit abscission from the pedicel AZ occur by similar mechanisms, the distribution of XTH and expansin epitopes were investigated by applying the same set of polyclonal antibodies to mature green (MG) and over ripe (OR) fruit pedicel sections, each representing a stage in which adhesion at the AZ was strengthened or loosened, respectively. In MG fruit pedicels, the intensities of XTH and expansin epitope labeling were relatively low (Figure 6). We detected vascular bundles of cell wall loosening protein epitopes, but they were not as intense (Figure 6). We did not observe AZ-specific labeling of any epitopes.

**FIGURE 7:**
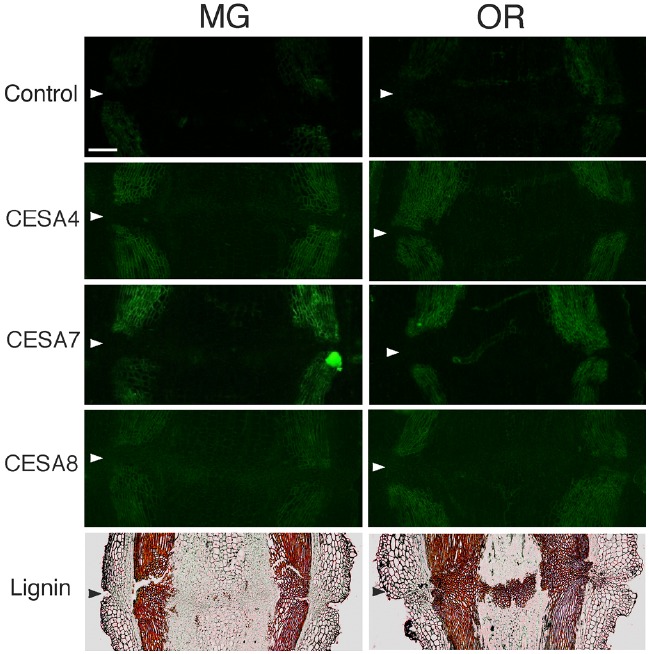
**Immunolocalization of CesA4, CesA7 and CesA8 epitopes in the AZs of mature green (MG) and over-ripe (OR) fruit pedicels.** Longitudinal sections of MG fruit pedicels, including the AZ, were stained with the anti-CesA4, 7, and 8 antibodies. Micrographs show the negative control without the first antibody step. Phloroglucinol staining for lignin of AZs in MG and OR fruit pedicels. Arrowheads indicate the AZs. Bar = 0.5 mm.

The immunolabeling pattern of OR fruit pedicels did not significantly differ from that of MG fruit pedicels (Figure 6); there were high labeling intensities of both XTH and expansin in the vascular bundles (Figure 7). The dramatic increase in expansin, specifically seen at the AZ, of the abscising flower pedicel was not observed in the loosened AZ of the OR fruit pedicel, nor was any AZ-specific labeling detected by immunostaining.

### Immunolocalization of Secondary Cell wall Synthase and Lignin Staining of MG and OR Fruit Pedicels

To determine the function of secondary CESA proteins (CESA4, CESA7, and CESA8) during fruit abscission, we used specific polyclonal antibodies to examine MG and OR fruit pedicel sections. CESA4, CESA7, and CESA8 are the most major marker enzymes for secondary cell wall synthesis (Carroll et al., 2012). The immunogens of the polyclonal antibodies of CESA4, CESA7, and CESA8 were produced from a recombinant peptide of cellulose synthase A catalytic subunit 4 (Q84JA6), 7 (Q9SWW6) and 8 (Q8LPK5), respectively^8^. Sequence similarity at the amino acid level of cellulose synthase A catalytic subunit 4, 7, and 8 are quite high compared with tomato CESA like genes (87, 91 and 88% identical, respectively), and there is one ortholog for the each CESAs in the tomato genome. Therefore, these commercial polyclonal antibodies of CESA4, 7, and 8 recognize CESA4, 7, and 8 of tomato with high probability. The results of immunostaining indicated no significant increase, decrease, or distributional change in cell wall epitopes in the AZs of loosening OR fruit pedicels. However, phloroglucinol staining was specifically observed at the AZs of the OR fruit pedicels (Figure 7). The lignin staining by phloroglucinol was not seen in the AZs of MG fruit pedicels (Figure 7), suggesting that the lignification was associated with an abscission-specific event.

## Discussion

We observed natural abscission at two different stages of fruit development in the tomato pedicel: in unsuccessfully pollinated flower pedicels during the time of fruit set, and at the end of fruit development in pedicels of fully ripened fruits. However, the actual shedding event seemed to occur differently in the two scenarios (Figure 1). In OR fruit, adhesion at the AZ weakened and became vulnerable to physical stress, but was kept intact until harvested by hand, whereas in unpollinated flowers, shedding occurred naturally. We previously reported that during floral abscission, a large increase was observed in LM15 labeling of xyloglucan, specifically at the AZ in the abscised pedicel. During fruit abscission, unlike in floral abscission, no AZ-specific deposition of cell wall polysaccharides was observed. However, high autofluorescence was seen in the AZ of overripe fruit pedicels, suggesting secondary cell wall synthesis and lignification of the AZ prior to fruit abscission (Iwai et al., 2013). Because of the small size and limited amount of AZ samples that can be examined, enzymatic analyses of cell wall modifying activities are quite difficult, therefore we visualized the presence of XTH, expansin, and cellulose synthase using immunohistochemistry.

To analyze the changes occurring within the AZ, cell-wall polysaccharides were visualized by immunolocalization. Previous research has identified a number of cell wall-degrading enzymes, including endoglucanases (Tucker and Milligan, 1991; Tucker et al., 1991; del Campillo and Bennett, 1996; Mishra et al., 2008), polygalacturonases (Kalaitzis et al., 1995, 1997; Gonzalez-Carranza et al., 2002, 2007), and XTHs (Campbell and Braam, 1999) that are involved in the abscission of tomato fruits. The levels of these enzymes increase during the abscission process; therefore, we focus on the cell wall and its remodeling, which may be the target or product of enzyme activity during abscission of the reproductive organ.

At the beginning of the study, we speculated that cell wall loosening through the abscission of the xyloglucan moieties by xyloglucan endohydrolase (XEH) activity might be important. However, during floral abscission, we observed a large 1 DPA peak in the polyclonal antibody labeling of XTH in the AZ, which then decreased (Figure 5). In addition, the intensity of LM15 labeling for xyloglucan epitopes increased dramatically, preferentially at the AZ (Figure 3). These results indicate that gradual xyloglucan accumulation is important for the abscission event. XTHs were not detected in last stage of abscission (yellowing). This result indicates that XTH protein is degraded in the relatively short period. Similarly the rapid turnover of XTH protein was reported during the expansion of tomato fruit (Catalá et al., 2000). LeEXT1 levels were high in young expanding fruit, exhibiting a peak at the fruit expansion stage, but decreased dramatically at the expanded fruit stage. Almost all XTHs studied to date have shown endotransglucosylase activity (Vissenberg et al., 2000; Baumann et al., 2007). The increase in xyloglucan endotransglucosylase (XET) action indicates active cell wall remodellings and reconstructions of the xyloglucan related cell walls in the AZ during the 1 DPA stage. It is likely that changes mediated by XET action may allow easier accessibility of the cell wall to other cell wall hydrolytic enzymes, thus accelerating abscission. Conversely, abscission is associated with an increase in cell size in *Arabidopsis* and citrus (Bleecker and Patterson, 1997; Agustí et al., 2009). XET action could be required for rearrangement of the cross-linking cell wall hemicelluloses during cell function is enlargement. This could lead to a decrease in cell wall mechanical strength, and hence abscission.

Expansin associated with the process of wall extension during cell growth (Li et al., 2003). It has, however, become clear that expansins also make a significant contribution to the process of fruit softening, which involves wall breakdown, rather than expansion. It has been observed that fruit softening and abscission share a number of features in common (Rose et al., 2003). *Arabidopsis* expansin, AtExp10, resulted in the expression of β-glucuronidase specifically at the base of leaf petioles and silique pedicels where they join the inflorescence stem. The authors proposed that this indicated a role of AtExp10 in abscission (Cho and Cosgrove, 2000). Expansins have functions that may increase disorder in cellulose crystals, making glucan chains more susceptible to hydrolysis (Cosgrove, 1999). In our data, AZ localize expansin epitopes increased dramatically and the highest levels of expansin were detected during the yellowing stage just prior to abscission (Figure 5), These results suggest that expansins may increase disorder in cellulose crystals, making the glucan chains more susceptible to hydrolysis. This suggests that expansins play a role in abscission by promoting the degradation of the linkage between cellulose and other components in the cell wall.

A stable level of xyloglucan may be important during rapid cell expansion, which is accompanied by rapid cell wall synthesis, and suggests functions related to XTH and expansin. Cell wall remodeling through rearrangement of the wall xyloglucans by the XET action of one to several XTHs might be essential determinants in the process of abscission. And expansin increase may be a trigger function of floral abscission.

In the case of leaflet abscission in *Sambucus nigra*, ethylene-promoted expansin was important for undergoing cell separation (Belfield et al., 2005). Likewise ethylene-responsive XTH was also important for petal abscission in rose (Singh et al., 2011). Therefore, ethylene might be important for floral abscission in tomato.

The immunostaining results from both MG and OR fruit pedicel AZs were similar, showing a relatively high intensity of XTH and expansin labeling of HG at the epidermis in and around the vascular bundles. During fruit development and formation, the pedicel itself, as well as the vascular bundles, thickens and its diameter increases, as can be seen by comparing flower and fruit pedicel micrographs (Figures 2–5). The strong XTH and expansin labeling in the MG fruit pedicels may be due to changes in the cell walls; possibly a new cell-wall network is created by bonding xyloglucan and cellulose. The labeling intensity of cell wall modifying proteins may reflect newly created cellulose-pectin binding that occurs via xyloglucan-based side chains that may lead to the development of stronger vascular tissue for delivering large amounts of water to the developing fruit.

Although strong labeling was observed in the vascular bundles and epidermis, no AZ-specific XTH and expansin epitope labeling was observed in either the MG or OR fruit pedicel AZs (Figure 6 and 7). The dramatic deposition of expansin in the flower AZ prior to abscission was not apparent in the OR fruit AZ (Figure 6 and 7). In our previous report, no AZ-specific LM6 and LM15 epitope labeling was observed in the MG or OR fruit pedicel AZs. It showed that the intensity of arabinan and xyloglucan labeling was low in both the MG and OR pedicels (Iwai et al., 2013). However, AZ-specific lignification was observed in the AZ of the OR fruit pedicel. These results suggest that lignification was due to secondary cell wall components, including lignin and other phenolics (Figure 7). However, AZ-specific secondary CESA proteins (CESA4, CESA7, and CESA8) were not detected during fruit abscission. These results suggest AZ-specific lignification might not be associated with normal secondary cell wall synthesis. Floral abscission following unsuccessful pollination is fundamentally different from that following fruit ripening. Floral abscission occurs by a remodeling involving the deposition of cell wall polysaccharides, whereas fruit abscission occurs through lignin deposition.

The results of our study suggest that floral abscission, determined by the successfulness of pollination, and fruit abscission that occurs post-ripening, are regulated by different mechanisms—floral abscission through the remodeling of cell wall polysaccharides, and fruit shedding through lignification.

### Conflict of Interest Statement

The authors declare that the research was conducted in the absence of any commercial or financial relationships that could be construed as a potential conflict of interest.

## Footnotes

1 www.plantprobes.net

2 www.agrisera.com/en/index.html

3 http://www.agrisera.com/en/artiklar/cesa4-cellulose-synthase-a-catalytic-subunit-4-udp-forming.html

4 http://www.agrisera.com/en/artiklar/cesa7-cellulose-synthase-a-catalytic-subunit-7-udp-forming.html

5 http://www.agrisera.com/en/artiklar/cesa8-cellulose-synthase-a-catalytic-subunit-8-udp-forming.html

6 http://www.biocompare.com/9776-Antibodies/2820012-anti-EXPB-2-antibody

7 http://www.antibodies-online.com/antibody/726770/anti-beta+(Expansin+EXPB2/)

8 www.agrisera.com product No. AS12 2582, AS12 2581, and AS12 2580.
